# Immunotoxicity of perfluorinated alkylates: calculation of benchmark doses based on serum concentrations in children

**DOI:** 10.1186/1476-069X-12-35

**Published:** 2013-04-19

**Authors:** Philippe Grandjean, Esben Budtz-Jørgensen

**Affiliations:** 1 Department of Environmental Medicine, University of Southern Denmark, Odense, Denmark; 2 Department of Environmental Health, Harvard School of Public Health, Boston, MA 02215, USA; 3 Department of Biostatistics, University of Copenhagen, 2100, Copenhagen, Denmark

**Keywords:** Benchmark dose, Developmental exposure, Immunotoxicity, Perfluorinated compounds, Risk assessment

## Abstract

**Background:**

Immune suppression may be a critical effect associated with exposure to perfluorinated compounds (PFCs), as indicated by recent data on vaccine antibody responses in children. Therefore, this information may be crucial when deciding on exposure limits.

**Methods:**

Results obtained from follow-up of a Faroese birth cohort were used. Serum-PFC concentrations were measured at age 5 years, and serum antibody concentrations against tetanus and diphtheria toxoids were obtained at age 7 years. Benchmark dose results were calculated in terms of serum concentrations for 431 children with complete data using linear and logarithmic curves, and sensitivity analyses were included to explore the impact of the low-dose curve shape.

**Results:**

Under different linear assumptions regarding dose-dependence of the effects, benchmark dose levels were about 1.3 ng/mL serum for perfluorooctane sulfonic acid and 0.3 ng/mL serum for perfluorooctanoic acid at a benchmark response of 5%. These results are below average serum concentrations reported in recent population studies. Even lower results were obtained using logarithmic dose–response curves. Assumption of no effect below the lowest observed dose resulted in higher benchmark dose results, as did a benchmark response of 10%.

**Conclusions:**

The benchmark dose results obtained are in accordance with recent data on toxicity in experimental models. When the results are converted to approximate exposure limits for drinking water, current limits appear to be several hundred fold too high. Current drinking water limits therefore need to be reconsidered.

## Background

Perfluorinated compounds (PFCs) have been in use for over 60 years in a wide array of applications. PFCs were first manufactured in the US from about 1947, with perfluorooctanoic acid (PFOA) and perfluorooctane sulfonic acid (PFOS) as primary products [1]. PFC was later found to contaminate ground and surface water, and PFOS was found to accumulate in freshwater fish [2]. These compounds possess a strong carbon-fluorine bond, which leads to persistence of the PFCs in the environment and the human body [2]. Thus, the high thermal, chemical and biological inertness that make the PFCs useful for many industrial purposes at the same time also generates an environmental hazard.

Serum-PFC analyses conducted by the Centers for Disease Control and Prevention (CDC) show that PFOS and PFOA are detectable in virtually all Americans [3], with children often showing higher serum concentrations than adults [4]. Analyses of paired samples of maternal serum and cord serum show that PFCs are transferred through the human placenta [5,6]. Due to global dissemination of PFCs, their serum concentrations in children and pregnant women even in the remote locations, such as the Faroe Islands [7], are similar to US levels. Exposures to some PFCs in the Faroes may occur primarily through marine diets [8]. Despite the extensive use of these compounds for many decades, and the persistence and cumulative properties of the PFCs, the toxicology data base is still incomplete and has allowed only preliminary risk assessments so far.

Using animal toxicity data, calculations of benchmark dose levels (BMDLs) have been carried out for a 10% deviation relative to control values (i.e., a Benchmark Response or BMR of 10%); they resulted in serum concentrations of 23 mg/L and 35 mg/L for PFOA and PFOS, respectively [9-11]. Toxicokinetic modeling and standard assumptions about water intake then allow derivation of acceptable drinking water levels [11,12]. So far, the U.S. Environmental Protection Agency (EPA) has issued a draft risk assessment of PFOA in 2005, but no final version has yet been published, nor has a Reference Dose (RfD) been defined. However, the EPA has issued provisional health advisories of 0.4 μg/L (400 ng/L) for PFOA and 0.2 μg/L (200 ng/L) for PFOS in drinking water [13]. Similarly, the Agency for Toxic Substances and Disease Registry concluded in its draft toxicological profile in 2009 that there was insufficient evidence at the time to develop a minimal risk level [1]. For chronic exposure, state authorities have issued limits for PFC concentrations in drinking the water, e.g., in Minnesota [14], where the limit for both PFOS and PFOA is 0.3 μg/L (300 ng/L). The limits were based on PFOS effects on the liver and thyroid, and PFOA effects on the liver, fetal development, reduction in red blood cell numbers, and immune system changes in experimental studies [11]. A lower guidance limit of 0.04 μg/L (40 ng/L) has been determined for PFOA by the state of New Jersey [15]. Other agencies, such as the European Food Safety Authority [16] have recommended similar exposure limits that relied on the same toxicology data while using different default assumptions.

PFC toxicity in animal models at first suggested the liver as a main target organ, but so far chronic toxicity data only in the rat have been published [1,12,17,18]. However, recent evidence suggests that toxicology outcomes used in derivation of exposure limits may not represent the most sensitive endpoints. Thus, interference with mammary gland development in mice with developmental exposure seems to occur at low exposures; benchmark dose calculations using a variety of models showed that a 10% BMR corresponded to a serum-based BMDL for PFOA of 23–25 μg/L (or ng/mL) [12,17]. This BMDL differs by a factor of 1,000 from the previously mentioned BMDL based on liver toxicity (i.e., 23 mg/L or 23,000 μg/L). Thus, current limits for PFOA in drinking water based on the latter value may not be as protective as intended, despite the use of uncertainty factors.

Likewise, immunotoxicity of PFCs has been demonstrated in rodent models, avian models, reptilian models, and mammalian and nonmammalian wildlife [19]. For example, in a commonly used mouse model, PFOA effects include decreased spleen and thymus weights, decreased thymocyte and splenocyte counts, decreased immunoglobulin response, and changes in specific populations of lymphocytes in the spleen and thymus. Reduced survival after influenza infection has also been reported as an apparent effect of PFOS exposure in mice [20]. Another study found that the lowest observed effect level (LOEL) for males corresponded to an average serum-PFOS concentration of 92 ng/g (about 94 μg/L), though 7-fold higher in females [21]. The LOEL serum concentration in males is similar to typical levels found in serum samples from subjects exposed to contaminated drinking water [22].

Given the concern about immunotoxicity as a possible critical effect [19] and the possibility of developmental toxicity [23], studies in child populations have recently focused on antibody responses to childhood immunizations as a clinically relevant parameter that reflects major immune system functions [24]. The subjects have all received the same doses of vaccine antigens at the same ages and can then be examined at similar ages, i.e., similar intervals after the most recent vaccination [25]. Our studies focused on the fishing community of the Faroe Islands [8], and these prospective population data [7] seem appropriate for calculating benchmark doses as a contribution to future risk assessments.

While benchmark dose calculations from toxicology data are fairly straightforward, using epidemiological studies can be more complicated due to the need for covariate adjustments [26]. In addition, decisions on dose–response models may be crucial, as a null exposure group is usually not available, thus requiring extrapolations beyond the exposure interval observed.

## Methods

A birth cohort in the Faroe Islands was recruited and consisted of 656 consecutive singleton births from late 1997 to early 2000. Prospective follow-up included 587 cohort members participated in one or both examinations at ages 5 and 7 years [7], of whom 460 participated on both examinations, and complete data with serum analyses were obtained for 431. As exposure indicator, we used the PFC concentrations in the child’s serum obtained at the clinical examination at age 5 years. The outcomes were the specific antibody concentrations against tetanus and diphtheria toxoids in serum at age 7 years. Of the PFCs, PFOS and PFOA showed the highest concentrations (Table 1), similar to levels reported from the US [3]. We also measured maternal pregnancy serum PFC concentrations, which showed strong negative correlations with antibody concentrations at age 5 years. However, we chose to focus on the PFCs in the child’s serum at age 5 and their uniformly negative associations with antibody levels at age 7, as these data apparently represented the greatest sensitivity to PFC exposure so far documented and were not confounded by exposures to other environmental chemicals. The dependence of the antibody concentrations on PFC exposures was determined by generalized additive models [27]. Written maternal consent was obtained, and the protocol was approved by the ethical review committee at the Faroe Islands and by the review board at the US institution.

**Table 1:** Characteristics of 431 Faroese birth cohort members with complete data from examinations at ages 5 and 7 years

**Variable**	**Result**
Girl, n (%)	223 (48.5)
Birth weight, mean (SD) g	3724 (505)
Birth weight ≤ 2500 g, n (%)	3 (0.7)
Age at 5-year examnination, mean (SD) years	5.0 (0.1)
Age at 7-year examination, mean (SD) years	7.5 (0.1)
Serum-PFOS concentration at age 5, ng/mL^a^	17.3 (14.1; 21.3)
Serum-PFOA concentration at age 5, ng/mL^a^	4.06 (3.33; 4.95)
Anti-tetanus concentration at age 7, IU/mL^a^	1.80 (0.75; 4.60)
Anti-diphtheria concentration at age 7, IU/mL^a^	0.80 (0.40; 1.60)

^a^ Median (interquartile range).

### Benchmark calculations

The data were analyzed as continuous variables in SAS version 9.2. Although a clinical cut-off level exists for antibody concentrations that represent long-term protection, this limit is somewhat arbitrary, and transformation of the continuous data to a dichotomous variable results in a loss of information.

Benchmark calculations were therefore based on regression models with antibody concentrations as dependent variables while PFC-concentrations were included as independent variables along with potential confounders sex, age and booster type at age 5 [7]. To achieve normally distributed residuals, antibody concentrations were log-transformed. Thus, we based models on the formula

$$\begin{array}{rcl} \log\left(\mathrm{antibody}\right) & = & \alpha_{0}+\alpha_{1}\times \mathrm{sex}+\alpha_{2}\times \mathrm{age}+\alpha_{3}\\ & & \times \mathrm{booster}\;\mathrm{type}+f\left(d\right)+\varepsilon , \end{array}$$

where d is the PFC concentration (PFOS or PFOA) measured at 5 years and f is the dose–response function satisfying f(0) = 0. We modeled the PFC-effect using a linear-dose response function [f(d) = β × d], a logarithmic model [f(d) = β × log(d + 1)] and the so-called K-power model [f(d) = β × d^K^, K > =1]. As the dose–response relationship at low doses may differ from the one at higher doses, we also used a piecewise linear model, which allowed for a difference in slopes at the median exposure. Calculations were carried out for PFOS and PFOA separately. Given their close correlations, it was not possible to include mutual adjustment in the models.

The BMD is the dose which reduces the outcome by a certain percentage (BMR) compared to unexposed controls [28,29]. Several different BMR values have been used in the past, and lower BMR levels are known to result in decreased BMD results, in part because the uncertainty increases [26]. By convention, a 10% BMR is often used for experimental toxicology data [28,29]. On the other hand, a decreased antibody response to vaccinations must be regarded as an important adverse effect, thus supporting the selection of a lower BMR. Thus, in human studies, a BMR of 5% is often chosen [29]. We therefore calculated BMD results for BMR values of 5% and 10%. An advantage of a log-transformed response is that BMD can be estimated independently of the confounders as the dose where the dose–response function is equal to log(1-BMR), i.e., the BMD, will satisfy the equation f(BMD) = log(1-BMR).

As the main result of the calculations, the benchmark dose level (BMDL) is defined as the lower one-sided 95%-confidence limit of the BMD. In the dose–response models with linear parameters (linear, log and piecewise linear models), the derivation of closed form expressions for the BMDL is straight forward [30]. Based on the estimated uncertainty in the parameter estimates, the lower confidence limit of the dose-effect function [f(d)] can be determined. The BMDL is given as the dose where this confidence limit is equal to log(1-BMR). For non-linear models, the BMDL was calculated using the (iterative) profile likelihood method. The fit of the models was based on minus two times the log-maximum likelihood function (−2 log(L)), where a smaller value indicates a better fit. The low dose fit was measured by calculating -2log(L) based on children with exposures in the lowest quartile.

**Figure 1:**
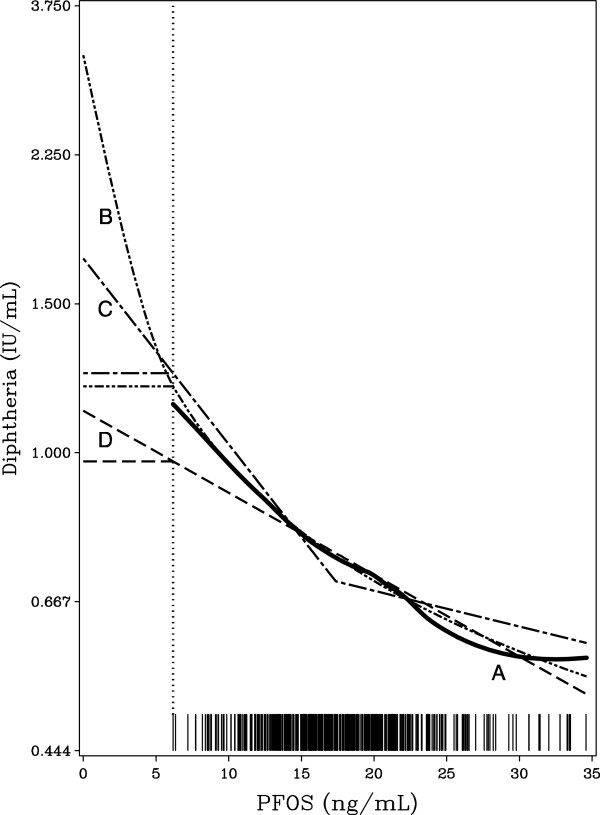
**Estimated dose–response functions for the relationship between PFOS and the diphtheria-antibody concentration. Curve A** is estimated as a generalized additive model. **Curve B** is the log-function, **C** is piecewise linear, and **D** is linear. The low-dose threshold models (see Table 3) assume a flat curve below the lowest observed dose indicated by the dotted vertical line, i.e., that a threshold exists at the lowest serum-PFOS concentration observed. The bars on the horizontal scale indicate the serum-PFOS concentration of each participating cohort member.

As a consequence of the relatively steep dose–response relationships, the BMDs were sometimes lower than the minimum observed exposure, and some results therefore depended on a part of the dose–response curve, for which the data does not hold any information. As a sensitivity analysis, we therefore developed a low-dose threshold version of each of the dose–response models used. Each of these models was identical to the original dose–response model within the observed dose range, but with a flat dose–response slope below the lowest dose observed (Figure 1).

## Results

Descriptive results are shown in Table 1. Children who participated in one clinical examination, but not the other, did not seem to differ in terms of exposure levels and antibody concentrations from those cohort subjects who participated in both examinations.

Generally, the log model yielded lower BMDs, but only for the PFOS did these results provide a (marginally) better fit than the linear slope (Table 2). The model-dependence was similar for tetanus and diphtheria antibody concentrations as outcome variables. When using the linear slope and a BMR of 5%, the BMDL was about 1.3 ng/mL and 0.3 ng/mL for PFOS and PFOA, respectively. The piecewise linear curve showed BMDL results about half the level of the linear dose–response curve, while the logarithmic curve showed even lower results. In the K-power model, the power parameter was estimated to one, and this model was therefore identical to the linear model. As expected, results were higher at a BMR of 10%.

**Table 2:** Benchmark results for postnatal PFC exposure (in terms of serum concentrations in ng/mL measured at 5 years) with vaccine antibody concentrations at 7 years as the outcomes

			**BMR = 5%**		**BMR = 10%**		**Fit (−2log(L))**	
**Outcome**	**Exposure**	Model*	**BMD**	**BMDL**	**BMD**	**BMDL**	**Full scale**	**Low dose**
Tetanus	PFOS	Linear	2.70	1.31	5.55	2.69	1719.81	313.78
		Log	0.13	0.07	0.29	0.14	1719.30	313.75
		Piecewise	1.45	0.56	2.98	1.16	1719.54	313.54
	PFOA	Linear	0.38	0.25	0.77	0.51	1712.43	391.53
		Log	0.07	0.04	0.14	0.09	1712.88	391.63
		Piecewise	0.52	0.16	1.07	0.34	1712.33	391.64
Diphtheria	PFOS	Linear	2.30	1.25	4.72	2.57	1656.86	314.00
		Log	0.11	0.06	0.24	0.13	1655.96	313.38
		Piecewise	0.98	0.49	2.01	1.01	1655.77	313.10
	PFOA	Linear	0.59	0.33	1.21	0.68	1656.15	362.37
		Log	0.10	0.06	0.22	0.12	1656.14	362.39
		Piecewise	0.48	0.17	0.99	0.34	1656.12	362.30

*K-power model was identical to the linear model.

All dose–response models had normally distributed residuals with a homogeneous scatter. The piecewise linear generally had the closest fit, but it was not significantly better than the alternative models. For the association between PFOS and the diphtheria antibody concentration, Figure 1 illustrates the agreement between the different models within the observed data range. The linear function is less steep at the low doses, which explains why this model yields higher benchmark results.

Using the low-dose threshold models with a flat dose–response below the lowest observed exposure levels, the BMDL results for the linear curve were about 5-fold higher than for the non-threshold curve (Table 3). The low-dose threshold results for both the piecewise and the logarithmic curves approximated those obtained using a linear slope.

**Table 3:** Results of sensitivity analyses using low-dose threshold models with no effect below the lowest observed exposures

		**BMR = 5%**		**BMR = 10%**	
**Outcome/Exposure**	**Model**	**BMD**	**BMDL**	**BMD**	**BMDL**
Diphtheria/PFOS	Linear	8.48	7.43	10.90	8.75
	Log	6.96	6.62	7.89	7.11
	Piecewise	7.16	6.67	8.19	7.19
Tetanus/PFOA	Linear	1.70	1.57	2.10	1.83
	Log	1.48	1.43	1.65	1.53
	Piecewise	1.85	1.49	2.40	1.66

Benchmark results for serum concentrations (in ng/mL) measured at 5 years in regard to vaccine antibody concentrations at 7 years. Results are given for the exposures and outcomes showing the lowest results in Table 1.

## Discussion

The present report presents the first benchmark dose results for human PFC exposure. It relies on serum-PFC measurements at age 5, and serum concentrations of specific antibodies two years later as clinically relevant measures of immune functions. The size and homogeneity of the study population and the high participation rate are major strengths [7]. The associations that appeared the strongest were selected for BMD calculations. Although this selection was not based on an *a priori* hypothesis and therefore could result in bias, structural equation model analyses suggest that the overall effects of PFCs on antibodies were stronger than most individual effects [7]. Concomitant exposure to PCBs did not cause any important confounding. We included age and sex as covariates, but they affected the results to a negligible degree only.

However, a weakness is the close correlation between PFOA and PFOS, which makes mutual PFC adjustment difficult. Structural equation models suggest that the joint effects of major PFCs were stronger than those that could be ascribed to single compounds [7], and it is therefore possible that each of the major PFCs contribute to the effects. Given the strong experimental support for immunotoxicity of both PFOA and PFOS [19], the BMD levels would seem to provide approximate levels of concern for human exposures.

The choice of dose–response models is known to result in different BMD results from epidemiological studies, where unexposed controls are often missing [26]. In the absence of prior knowledge regarding the shape of the curve, we used two common curve shapes (linear and logarithmic) to explore the dependence of the data on these two assumptions. The two curves fit the data equally well, and no statistical justification is therefore available for choosing one set of results above the others. The linear curve is often used as a default, and we therefore further examined a model with a piecewise linear shape and one with a flat slope below the lowest observed level of exposure. For each of the two PFCs, these sensitivity analyses showed that the BMDL results remained low. As anticipated, the 5% BMR results in BMDL values somewhat below those for 10%, but differences between the curve shapes were not smaller at an increased BMR.

The vaccine-specific antibody concentrations used in our recent study [7] are thought to represent sensitive immunotoxicity parameters. Other clinical outcome measures may be less sensitive. For example, hospitalization of 363 children up to an average age of 8 years for infectious diseases (such as middle ear infection, pneumonia, and appendicitis) was not associated with PFOS and PFOA concentrations in serum from pregnant women from the Danish National Birth Cohort [31]. Multiple social, demographic and other factors may have affected these results, and hospitalization does not seem to be a sensitive or appropriate test of the presence of immune system dysfunction. In adults exposed to PFOA through contaminated drinking water, the serum-PFOA concentration was associated with lower serum concentrations of total IgA, IgE (in females only), though not IgG [32]. Although confirmation from other human studies is therefore lacking so far, experimental studies offer support that specific immunoglobulin concentrations may be sensitive indicators of immune system dysfunctions [19].

Interaction with peroxisome proliferator-activated receptors (PPARs) may be involved in the immunotoxic mechanisms [1,19]. While human PPARα expression is significantly less than that of rodents, current evidence suggests that both PPARα-dependent and -independent pathways may be relevant to PFC immunotoxicity [33]. In human white blood cells *in vitro*, mechanistic studies of PFC-induced suppression of cytokine secretion demonstrated that PPARα activation was involved in the PFOA-induced immunotoxicity, while other pathways appeared responsible in regard to the effects of PFOS [34]. White blood cells from human volunteers showed effects at PFOS concentrations in the medium of 0.1 μg/mL (100 ng/mL), which was the lowest concentration tested [35]. This level is similar to concentrations seen both in affected male mice [21] and in subjects exposed to contaminated drinking water [22].

Based on both experimental and human studies, an approximate BMDL of 1 μg/L would seem to be an appropriate order of magnitude for calculation of exposure limits for the PFCs. As the BMDL assumes equal sensitivity within the population studied, current guidelines [28,29] require that the BMDL be divided by an uncertainty factor of 10 to take into account the existence of subjects with increased vulnerability. A concentration of about 0.1 ng/mL could then be used as the serum-based RfD for the PFCs (somewhat higher for PFOS and lower for PFOA).

Using mammary gland development as a sensitive outcome in experimental studies [17], a BMDL of about 23 ng/mL serum was calculated for PFOA [12]. Taking into account interspecies differences in vulnerability and using a total uncertainty factor of 30, an RfD of 0.8 ng/mL serum would be derived from this BMDL. Thus, although referring to a different endpoint, this calculation is in good accordance with the one estimated from our epidemiological data.

A serum-based RfD less than 1 ng/mL for PFOS and PFOA would be below most concentrations reported in recent studies [3,7,31]. Importantly, estimated RfD values below 1 ng/mL are at least 100-fold below those used for calculation of current water contamination limits. PFOA concentrations in drinking water are known to correlate with the serum concentrations of long-term residents in Ohio and West Virginia at an approximate ratio of about 1:100 [12,15,36]. Thus, from these data, a serum-based RfD of 0.1 ng/mL can be translated to a water concentration of 1 ng/L, or 0.001 μg/L (assuming that no other sources contributed to the PFOA exposure). The current EPA limit for this PFC is 300-fold higher. Thus, the recent evidence on PFC immunotoxicity in humans and toxicity in animal models suggests that current limits for drinking water contamination are too permissive and must be decreased substantially.

## Conclusions

BMDL results were about 1.3 ng/mL serum for PFOS and 0.3 ng/mL serum for PFOA at a benchmark response of 5%. Lower values were obtained with the logarithmic curve, and higher results with a larger benchmark response. The BMDL results are in accordance with recent data on toxicity in experimental models. When converted to approximate exposure limits for drinking water, current limits appear to be several hundred fold too high. Current drinking water limits therefore need to be reconsidered in the light of the observed immunotoxicity associated with PFC exposure.

## Abbreviations

BMD: Benchmark dose; BMDL: Benchmark dose level, i.e., the lower 95% confidence limit for the BMD; BMR: Benchmark response; EPA: Environmental Protection Agency; LOEL: Lowest observed effect level; log: Natural logarithm; PFC: Perfluorinated compound; PFOA: Perfluorooctanoic acid; PFOS: Perfluorooctane sulfonic acid; PPAR: Peroxisome proliferator-activated receptor; RfD: Reference Dose.

## Competing interests

PG is editor-in-chief of this journal, but was not involved in the editorial handling of this manuscript. The authors declare that they have no competing interests.

## Authors’ contributions

PG performed the literature review and drafted the manuscript. EBJ carried out the benchmark analyses and commented on the interpretation. Both authors contributed to, read, and approved the final version.
